# A Method for Whole Brain *Ex Vivo* Magnetic Resonance Imaging with Minimal Susceptibility Artifacts

**DOI:** 10.3389/fneur.2016.00208

**Published:** 2016-11-29

**Authors:** Anwar S. Shatil, Kant M. Matsuda, Chase R. Figley

**Affiliations:** ^1^Biomedical Engineering Graduate Program, University of Manitoba, Winnipeg, MB, Canada; ^2^Neuroscience Research Program, Winnipeg Health Sciences Centre, Kleysen Institute for Advanced Medicine, Winnipeg, MB, Canada; ^3^Department of Pathology, University of Manitoba, Winnipeg, MB, Canada; ^4^Department of Radiology, University of Manitoba, Winnipeg, MB, Canada; ^5^Division of Diagnostic Imaging, Winnipeg Health Sciences Centre, Winnipeg, MB, Canada; ^6^Department of Psychological and Brain Sciences, Johns Hopkins University, Baltimore, MD, USA

**Keywords:** *ex vivo*, fixation, formalin, human brain, MRI, neuroimaging, postmortem

## Abstract

Magnetic resonance imaging (MRI) is a non-destructive technique that is capable of localizing pathologies and assessing other anatomical features (e.g., tissue volume, microstructure, and white matter connectivity) in postmortem, *ex vivo* human brains. However, when brains are removed from the skull and cerebrospinal fluid (i.e., their normal *in vivo* magnetic environment), air bubbles and air–tissue interfaces typically cause magnetic susceptibility artifacts that severely degrade the quality of *ex vivo* MRI data. In this report, we describe a relatively simple and cost-effective experimental setup for acquiring artifact-free *ex vivo* brain images using a clinical MRI system with standard hardware. In particular, we outline the necessary steps, from collecting an *ex vivo* human brain to the MRI scanner setup, and have also described changing the formalin (as might be necessary in longitudinal postmortem studies). Finally, we share some representative *ex vivo* MRI images that have been acquired using the proposed setup in order to demonstrate the efficacy of this approach. We hope that this protocol will provide both clinicians and researchers with a straight-forward and cost-effective solution for acquiring *ex vivo* MRI data from whole postmortem human brains.

## Introduction

Magnetic resonance imaging (MRI) has become one of the most commonly used medical imaging modalities among human neuroscientists due to its ability to non-invasively visualize and quantify various anatomical and microstructural characteristics of the brain. However, its non-destructive properties also make MRI a popular method for studying postmortem human brains *ex vivo*. Compared to *in vivo* scanning, *ex vivo* imaging (e.g., of autopsy brains) allows for extremely long MRI experiments that are free of subject motion and other sources of physiological noise, and therefore, higher spatial resolution and signal-to-noise ratios than are achievable *in vivo*. For these reasons, imaging postmortem human brains with various MRI techniques – e.g., MR microscopy, diffusion tensor imaging (DTI), high angular resolution diffusion imaging (HARDI), diffusion spectrum imaging, magnetization transfer imaging (MTI), multi-component T_2_-relaxation myelin water imaging (MWI), etc. – has become a popular option (1–12).

There are, however, a few additional challenges in *ex vivo* MRI that make it more complicated than *in vivo* imaging. For example, postmortem brains will degrade due to bacteria and autolysis unless chemical fixatives are used for preservation (13). Due to its efficacy and availability, formalin is the most common fixative for tissue preservation (14–16). However, it is known that fixatives alter various MRI properties, as observed in previous studies (11, 12, 17, 18). For example, postmortem interval (PMI) (7, 17, 19) – i.e., the amount of time between patient death and initiation of tissue fixation – is associated with tissue decomposition (2); and scan interval (SI) (1) – i.e., the amount of time that tissue has been immersed in fixative at the time of MRI scanning – is hypothesized to cause acidity (20), dehydration (19), and protein cross-linking (13, 21). It should be noted that both PMI and SI alter various MRI properties, including: T2-relaxation, proton density (PD) measurements, fractional anisotropy (FA), apparent diffusion coefficient (ADC), and mean diffusivity (MD) (10–13, 18, 22–25). However, although these are important topics that should be considered in any postmortem MRI experiments (and likely warrant further investigation in their own right), they are beyond the scope of the current manuscript.

Instead, the intended goal of our protocol is to deal with the other major challenge associated with postmortem, *ex vivo* MRI scanning – namely, image artifacts that are often caused by magnetic susceptibility interfaces or magnetic susceptibility boundaries. *In vivo*, the brain is surrounded by cerebrospinal fluid (CSF) and other tissues (including the meninges, skull, and scalp), which create a stable and relatively homogeneous magnetic environment. However, once the brain is removed from this environment, large image artifacts can result from the magnetic susceptibility differences that occur at air–tissue boundaries (26–29). One potential solution to this problem is to scan postmortem brains *in situ*, before removing them from the skull (30), but this has obvious limitations and is either impractical or impossible in many situations. Another more common approach to mitigate magnetic susceptibility distortions has been to remove the brains and scan them *ex vivo* in a proton-free fluid called Fomblin (a chemically inert perfluoropolyether fluorocarbon; Solvay Solexis, Inc.), which produces no MRI signal but has a similar magnetic susceptibility to tissue (6, 7). However, although this is arguably the “gold-standard” *ex vivo* imaging approach, there are still several drawbacks. For example, Fomblin is very expensive (>$500 Canadian dollars per liter), and it is not readily available in most pathology labs or MRI centers – making it difficult to obtain (especially in quantities necessary for immersing whole human brains). Furthermore, Fomblin is difficult to completely remove from the surface of the specimen due to its oil-like properties, and this may interfere with subsequent brain fixation, embedding, and/or histological staining.

Therefore, we have developed an alternative approach that involves scanning *ex vivo* human brains in a MRI-compatible container that is completely filled with either water or formalin solution (i.e., in a magnetic environment similar to *in vivo* conditions), while taking special precautions to eliminate air bubbles from both the brain and the container. We have found, through trial and error, that the easiest and most effective method to completely eliminate bubbles is to completely immerse the MRI-compatible container in water or formalin, place the brain inside, and gently agitate to remove small surface bubbles, and then secure the container lid while completely submerged. In order to document our procedures (and hopefully pass our knowledge on to other researchers), our optimized protocol is described below in detail, and examples of resulting brain images are shown to demonstrate the efficacy of this approach.

## Materials and Equipment

The names and descriptions of all necessary materials are provided in Table 1, and optional materials (that are necessary for scanning in formalin) are listed in Table 2.

**Table 1:** **Name of necessary materials**.

Materials	Company	Comments/description
1-gallon (3.8 L), wide-mouth, water jug	Coleman	MRI-compatible brain container with interior/exterior dimensions of ~5.75/8.00″ (W) × 5.75/8.25″ (D) × 9.75/11.75″ (H). (Available on Amazon or at most camping/outdoor retailers and department stores.)
Small bottle or tube of plastic epoxy	Any	To permanently seal the spout and any other openings on the lid of the MRI-compatible brain container. (Available on Amazon or at most hardware stores.)
A medium sized, water-tight, rectangular, plastic bucket	Any	Water/formalin overflow bucket. Interior dimensions should be at least as wide, approximately twice as deep, and ~9″ taller than the brain container (i.e., >8″ × ~16″ × ~22″). (Available on Amazon or at most department stores.)
Minimal expansion polyurethane insulating foam	Dow Chemicals Great Stuff™ Gaps and Cracks Insulating Foam Sealant	To fill the voids between the water/formalin overflow bucket and the MRI-compatible brain container. For full product details, please refer to the Canadian Construction Materials Center product report (CCMC 13074-L). (Available on Amazon or at most hardware stores and department stores.)
5-lb barbell weight plate	Any	To keep the MRI-compatible brain container submerged in the overflow bucket during water/formalin filling. (Available on Amazon or at most sporting goods stores and department stores.)
Duct tape	Any	To temporarily attach the 5-lb weight to the bottom of the MRI-compatible brain container during the water/formalin filling procedure. (Available on Amazon or at most hardware stores.)
Extra-large garbage or yard waste bags	Any	To place between the overflow bucket and the MRI-compatible brain container during water/formalin filling. Also to place around the MRI-compatible brain container in the MRI system in case of small drips/leaks from the lid. (Available on Amazon or at most grocery stores and department stores.)
Cotton batting	Any	To place inside the top and bottom of the MRI-compatible container (in order to pad the brain specimen). (Available on Amazon or at most sewing/fabric stores and department stores.)
Latex or nitrile surgical gloves	Any	To wear while handling brain specimens. (Available on Amazon or at department stores.)
Lab coats (or surgical gowns)	Any	To wear while handling brain specimens. (Available on Amazon or from most chemical supply companies.)
Multi-contrast MRI fiducial marker	Any (e.g., Beekley Medical MR-SPOTS Packets™)	To correctly identify right/left hemispheres in subsequent MRI data. (Available upon request in most radiology departments and MRI centers, but can also be purchased from a local medical supply company.)
Either stitches or string (and possibly a hemostat or tweezers to tie)	Any	To attach the fiducial marker to remaining dura matter or cerebral vein. (Available in any pathology department.)
Scissors	Any	To cut duct tape, stitches, yard waste bags, etc. (Available on Amazon, in most department and hardware stores, or in most pathology departments.)
Human MRI system and head coil (additional flex coil optional)	Any	To acquire whole-brain, *ex vivo* MRI data. (Need to request access from local radiology department or MRI facility.)
Small foam pad or folded sheet	Any	To place between posterior elements of the head coil and the MRI-compatible brain container (in order to hold the container securely in place and reduce vibrations during scanning). (Available in any MRI facility.)

**Table 2:** **Name of optional materials (if using formalin)**.

Materials	Company	Comments/description
10% phosphate-buffered formalin solution	Any	pH-neutral solution for histological tissue (brain) fixation. [Available in most pathology departments, but can also be ordered from Sigma-Aldrich (Product ID: HT501128-4L).]
Formaldehyde spill response kits (2×)	Safetec	To safely manage and clean up small formalin spills. [Available from Fisher/Thermo Scientific (Product Name: Safetec Formaldehyde Spill Response Kit; Product ID: 19-314634).]
Fume hood	Any	For safety reasons, formalin solution should only be poured/handled in a well-maintained fume hood. (Available in most pathology departments and other “wet lab” spaces.)
Face shields	Any	For safety reasons, face shields and other protective clothing should always be worn while pouring/handling formalin solution. (Available in most pathology departments, but can also be purchased on Amazon.)
Chemical-resistant, shoulder-length gloves	Any	For safety reasons, chemical-resistant gloves and other protective clothing should always be worn while pouring/handling formalin solution. (Available in most pathology departments, but can also be purchased on Amazon.)
Baritainer (for chemical waste disposal)	Any	For discarding excess formalin solution. (Available in most pathology departments, but can also be purchased on Amazon.)
Formalin-neutralizing solution	Any	For neutralizing excess formalin solution. [Available in most pathology departments, but can also be purchased through Fisher/Thermo Scientific (Product Name: Formalex™; Product ID: 3120131).]
Heavy-duty (4 or 6 mil) polypropylene tubing (12″ wide)	Any	For placing around the MRI-compatible brain container before putting it in the MRI system (in case of small drips/leaks from the lid). (Available in most pathology departments, but can also be purchased on Amazon.)
Industrial heat impulse sealer (at least 12″ wide)	Any	For sealing the polypropylene tubing around the MRI-compatible brain container before putting it in the MRI system (in case of small drips/leaks from the lid). (Available in most pathology departments, but can also be purchased on Amazon.)

Before working with any new materials, manufacturers’ instructions [and material safety data sheets (MSDS), if applicable] should be carefully reviewed for safe handling, application, disposal, and spill cleanup procedures.

## Stepwise Procedures

Please note that all experimental procedures reported herein were carried out with proper consent and prior approval from The University of Manitoba Health Research Ethics Board.

### Preparation Steps (To Be Completed before Obtaining an *Ex Vivo* Brain)

1.1. Procure a wide-mouth, MRI-compatible plastic container with an air- and water-tight screw-on lid that is large enough to hold a whole human brain, but small enough to fit inside a MRI head coil.^1^ We have had excellent results using 1-gallon (3.8 L) Coleman Water Jugs (model No. 3000000865; exterior dimensions: 8″ × 8.25″ × 12.8″), which: (1) comfortably accommodated full-sized adult human brains, (2) were MRI-compatible, (3) fit inside a standard 12-channel Siemens head coil, and (4) did not produce any noticeable image artifacts.
1.2. If the lid of the MRI-compatible container has an opening or spout, this should be permanently sealed with plastic epoxy to avoid any accidental leaks or spills in subsequent steps. Please note that proper safety precautions should be followed while mixing and handling epoxy (e.g., working in a well-ventilated area and wearing disposable latex/nitrile gloves and safety goggles to avoid contact with your skin or eyes).
1.3. Obtain a rectangular, water-tight plastic bucket that is at least as wide, approximately twice as deep, and at least 9″ taller than the brain container described above in Section 1.1. This will give sufficient room for the experimenters to place the smaller container into the larger bucket (and open/close the lid) inside of the larger bucket.
1.4. *(Note: this step is only necessary for scanning ex vivo brains in formalin during subsequent steps, and is not necessary if scanning in water or saline.)* Wrap the small, MRI-compatible brain container described in Section 1.1 (without brain or formalin in it) with a large polyethylene (garbage/yard waste) bag, and place it inside the larger bucket. Then spray one layer (~3–4″ deep) of expanding polyurethane-based insulating foam sealant into the bottom of the larger bucket (i.e., around the smaller polyethylene-wrapped container) and wait for the polyurethane foam to fully expand and cure (~3–4 h) before adding another layer. Repeat this process until the expanding polyurethane foam has filled the larger bucket to a depth of 1–2″ below the top of the smaller polyethylene-wrapped brain container (approximately three layers total × 3–4 h/layer = 9–12 h). In this way, the brain container serves as its own mold, while the expanding foam insulation fills the voids between the smaller and larger buckets (i.e., to reduce the amount of wasted formalin in subsequent steps). The polyethylene bag is necessary in order to get the brain container out of the larger bucket after the foam dries and hardens. For the expanding polyurethane foam insulation, we used Great Stuff ™ Gaps & Cracks Insulating Foam Sealant (Dow Chemicals Product Number: 157911). As noted, it is highly recommended that the sealant be applied in layers in order to give ample time to expand and dry between subsequent rounds of application. Also, proper safety precautions should always be used while handling the polyurethane foam insulation (e.g., working in a well-ventilated area and wearing disposable latex/nitrile gloves and safety goggles to avoid contact with your skin or eyes). Then, once the shape is molded and the expanding foam has completely hardened, remove the polyethylene-covered brain container from the larger bucket. The whole objective of this step is to occupy volume inside of the large bucket so that less formalin will be wasted while filling the smaller bucket in subsequent steps.

### Obtaining Whole Postmortem Brain Specimens

2.1. Before procuring any *ex vivo* brain samples, it is essential to obtain proper research ethics approval and coordinate with local neuropathologists to acquire postmortem materials in an ethical and timely fashion.
2.2. Discuss all study inclusion/exclusion criteria so that the neuropathologists can screen and select appropriate cases based on clinical history, cause of death, etc.
2.3. If possible, it is advised that the MRI researcher wait in (or immediately outside of) the autopsy room in order to minimize the interval between brain removal and formalin immersion and/or the initial MRI scan.

### Pre-Imaging Steps (To Be Completed Immediately before Imaging Experiments)

**Figure 1:**
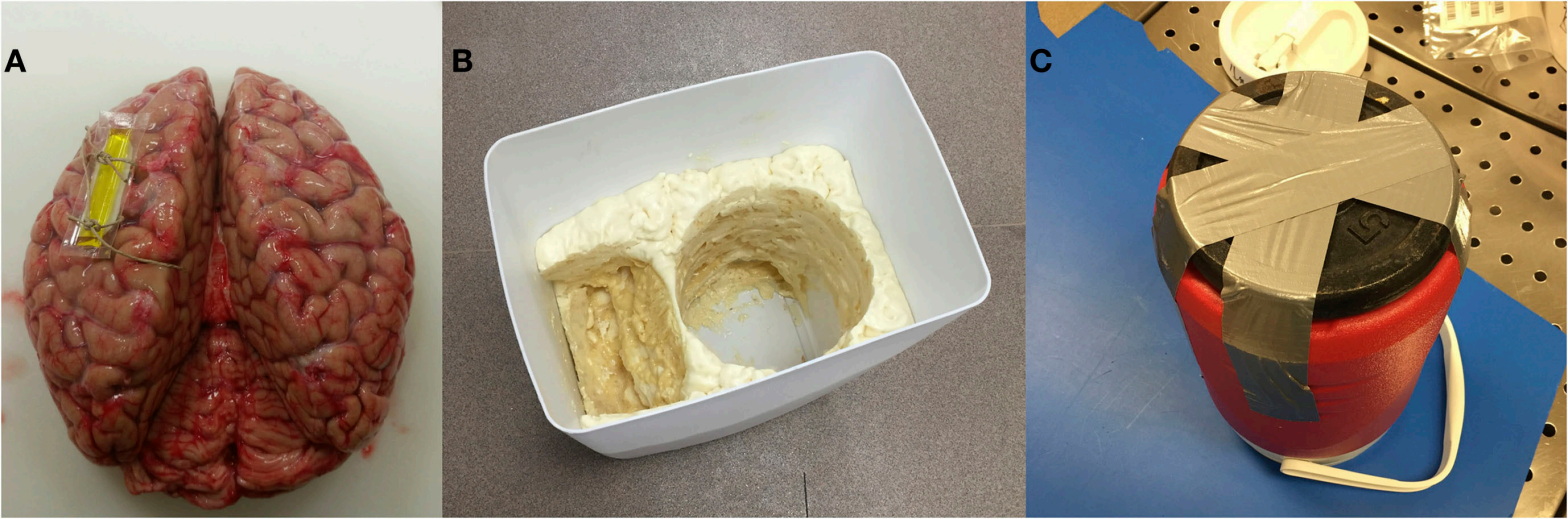
**(A)** Brain with a marker in the left hemisphere to detect left-right sides of the brain during image acquisition/processing; **(B)** a polyurethane-based insulating foam sealant sprayed inside the custom bucket to place the container and lid; **(C)** weight attached under the container to restrain it from floating.

**Figure 2:**
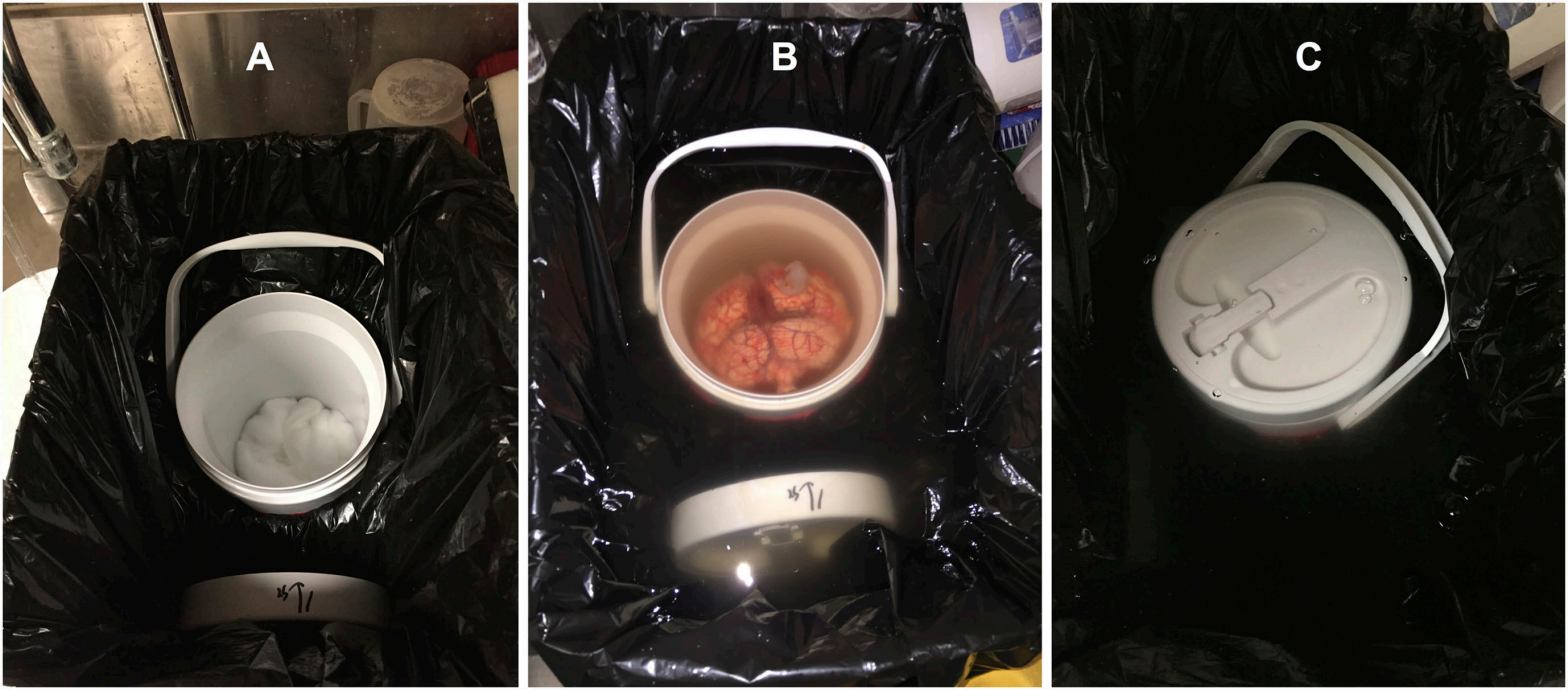
**(A)** Container and lid placed in the large bucket with a polythene bag wrapped around with some cotton in the bottom; **(B)** brain inside the bucket with formalin flowing over; **(C)** the container with closed lid under the formalin.

3.1. Attach a MRI-specific fiducial marker on either the left or right hemisphere (and note the location) to enable proper orientation of the brain during subsequent image acquisition (Figure 1A). The fiducial marker can typically be secured with one to two stitches around a flap of remaining dura matter (if present) or one of the major superficial cerebral veins.
3.2. Make sure that the larger bucket from steps 1.3 and 1.4 above – i.e., without or with the expanding foam insulation molding, depending on whether the subsequent preparation will be performed using water or formalin – is available for subsequent steps (Figure 1B).
3.3. Attach a 5-lb barbell weight plate to the bottom of the small, MRI-compatible brain container with duct tape (Figure 1C). The diameter should be less than or equal to the diameter of the container. Because the container is made of plastic and tends to float in water/formalin, attaching a weight will keep the container submerged. Then wrap the container (with the weight attached) in a large polythene (garbage or yard waste) bag and place it inside the corresponding mold in the larger bucket.
3.4. Cut two pieces of thick cotton and place one of them inside the bottom of the brain container to create a protective layer that is ~1–1.5″ thick (Figure 2A). The second piece of cotton will be used later to protect the brain from the lid (and will be added in step 3.11, before the lid of the container is secured).
3.5. Slowly pour water or formalin into the small, MRI-compatible brain container, and continue filling until the water/formalin level overflows into the larger container and is ~1″ above the top of the smaller container. Note: for this study, 10% phosphate-buffered formalin solution was used, which is widely available for routine histopathology (32). Caution: proper safety precautions should always be strictly adhered to while handling/pouring formalin (e.g., working in a fume hood while wearing closed-toe shoes, long pants, a lab coat or surgical gown, a face shield, and shoulder-length, chemical-resistant gloves), and a formaldehyde spill response kit (e.g., Fisher Scientific Product Number: 19-314634) should be readily accessible.
3.6. Squeeze the cotton at the bottom of the MRI-compatible container under the water/formalin for ~1 min to get rid of any air bubbles, and then splay it out in the bottom of the container. A freshly extracted brain is jelly like and can be easily deformed, so using cotton at the bottom of the container minimizes the risk of tissue damage.
3.7. Very gently, place the brain inside of the small, MRI-compatible brain container (i.e., carefully with both hands). In our experience, the orientation of the brain in the container does not matter, so either the anterior or posterior portion of the brain can be oriented toward the lid. Nonetheless, we recommend being consistent across subjects and/or time-points within a study. Note: in order to reduce the chances of having large air bubbles trapped in the lateral ventricles, water or formalin can be injected with a syringe directly into the ventricles (e.g., through the occipital lobe) to displace trapped air. However, it is highly recommended to perform this step only after consultation with the collaborating neuropathologist (i.e., to avoid compromising or interfering with subsequent clinical examinations, etc.).
3.8. Continue pouring water or formalin until the level is at least a few inches (i.e., greater than the depth of the lid of the MRI-compatible brain container) above the opening of the brain container (Figure 2B). Note: especially if using formalin, the experimenter should use his/her judgment to estimate the minimum amount of overflow necessary to complete the remaining steps and seal the container beneath the fluid level. This will minimize formalin waste, and more can always be added later if necessary.
3.9. The brain should then be slowly and gently agitated by hand (e.g., rotated left/right on its anterior/posterior axis) – while constantly submerged within the MRI-compatible container – for 2–3 min in order to ensure that no air bubbles are trapped along the cortical surface or under the cerebellum. The experimenter should use their judgment to agitate the sample enough to remove any visible bubbles, while ensuring that the brain specimen is not damaged in the process.
3.10. The second piece of cotton should then be submerged in the water or formalin, squeezed for ~1 min to remove any air bubbles, and then splayed out (while constantly submerged) over the brain at the top of the MRI-compatible container.
3.11. The lid of the MRI-compatible container should then be completely submerged and manipulated (if it has not been already) to remove any bubbles before closing it tightly beneath the level of the water or formalin (Figure 2C). Make sure that no cotton is stuck in the threads, and that the lid is not cross-thread before tightening. Note: screwing the lid on tightly is essential to prevent leaks in later steps; however, over-tightening may strip or distort the threads, so do not apply too much force.
3.12. After ensuring that the lid is secure, slowly and gently pull the MRI-compatible container out of the water or formalin in the larger bucket (i.e., using the handle if possible). Then detach the duct tape and the weight from the bottom and use paper and/or cotton towel to completely dry the exterior of the container before examining for any potential leaks (around the lid, or otherwise). Note: if leaks are identified, check to ensue that the lid is tight (but not over-tight), and if leaks persist, re-submerge the entire container, remove the lid, and try again (ensuring that no cotton gets caught between the threads).
3.13. Especially, if formalin was used to fill the brain container, it is recommended to heat-seal the entire MRI-compatible container inside of a heavy-duty (4 or 6 mil) polypropylene bag or tubing to catch any potential drips or minor leaks (and to act as a safeguard in the unlikely event that the container is dropped or damaged during transport). Make sure to squeeze the bag before sealing to reduce the amount of air trapped inside the bag as much as possible. If too much air is in the bag, it may not fit in the MRI head coil in later steps. Note: carefully check the sealed bag and, if in doubt, either repeat the previous step or add another layer of heat-sealed polypropylene tubing.
3.14. Finally, the brain container (whether heat-sealed in polypropylene bags/tubing or not) should be placed in two layers of large polyethylene (garbage/yard waste) bags – with the ends tied securely – to add an additional safeguard from potential drips or leaks during transport and/or MRI scanning. Again, make sure to squeeze as much air out of the bags as possible before tying (so that everything will fit inside of the MRI head coil).
3.15. Following step 3.14 above, the brain specimen is ready to be transported to the MRI facility, placed in the head coil, and scanned using the desired MRI pulse sequences.
3.16. The water or formalin waste (still in the larger bucket following step 3.13 above) can either be saved for later or properly disposed of. Note: water can likely be poured down any sink or autopsy table in the pathology department (check with a pathologist or pathology technologist), but formalin will need to be disposed of more carefully (see steps 3.17–3.19 below).
3.17. If formalin was used, it should be poured slowly and carefully (using a funnel) out of the polyethylene bag (in the large bucket) into a chemical Baritainer that has been specifically marked for formalin waste. Once there is very little formalin left in the polyethylene bag, carefully remove it from the large container and cut one of the bottom corners with scissors to drain any remaining formalin into the Baritainer. Then add the proper amount of formalin-neutralizing compound to the Baritainer and wait the specified amount of time before discarding the neutralized formalin. Note: the duct tape, polyethylene bags, and any paper towels, etc., with formalin on them should be placed into a garbage bag (which should then be securely tied) before removing from the fume hood and placing into an appropriate disposal bin (check with a pathologist or pathology technologist).
3.18. Leave the large bucket, 5-lb weight and shoulder-length, chemical-resistant gloves in the fume hood until the formalin has completely evaporated.
3.19. Finally, remove latex/nitrile gloves, safety glasses or face shield, and lab coat or surgical gown, and wash hands thoroughly.

### Recommended Setup for MRI Scanning

**Figure 3:**
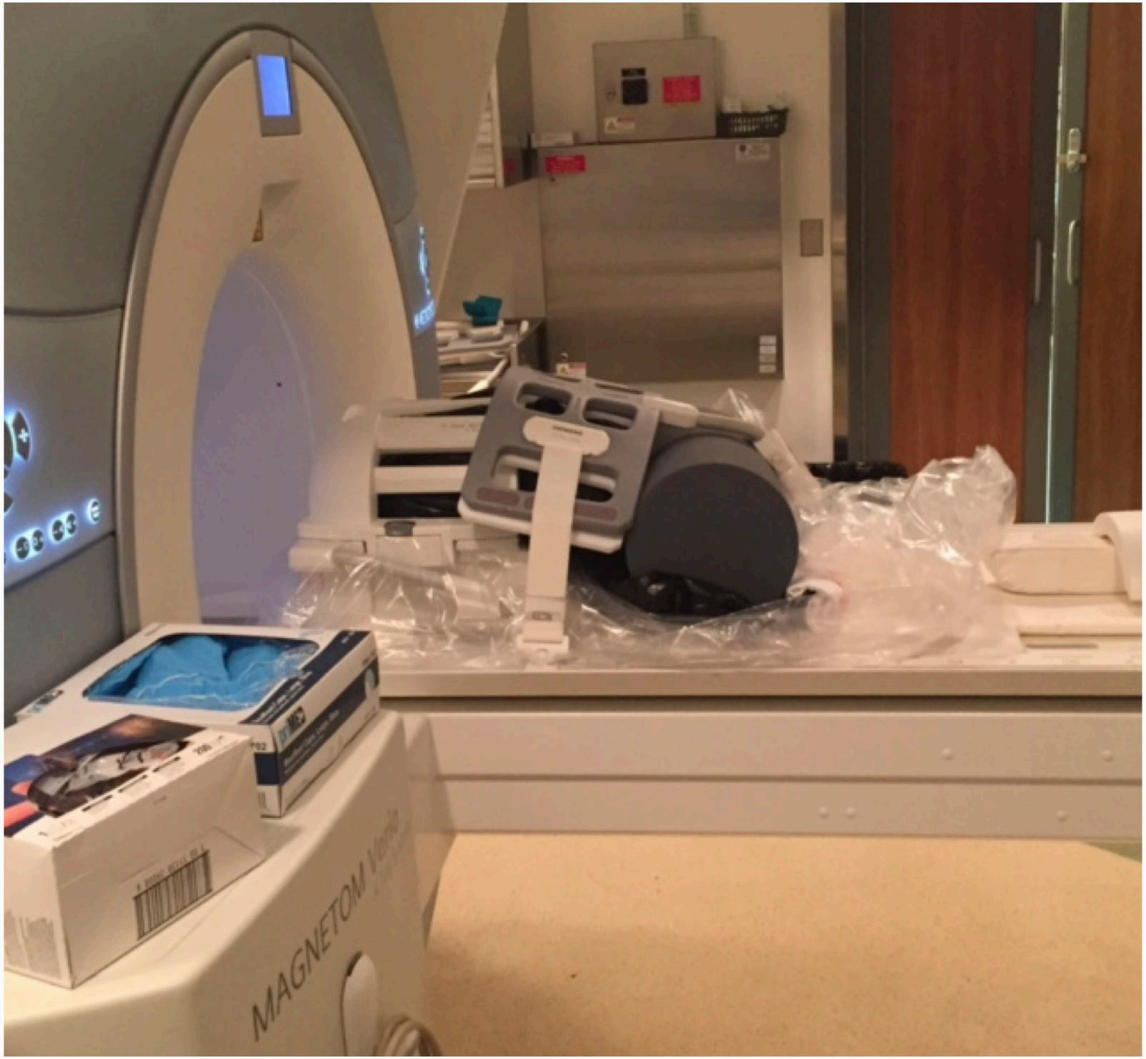
**Setup at the MRI facility with a 12-channel head coil and a 4-channel (knee) flex coil**. Cylindrical foam padding was placed under the MRI-compatible container to add stability and minimize vibrations during MRI scanning.

4.1. If using a two-part head coil for scanning, install the posterior elements of the head coil.
4.2. Out of an abundance of caution, we recommend taping a large polyethylene sheet or bag over top of the posterior head coil elements and patient table in case of small drips or leaks. Note: although the possibility of a leak or spill is extremely unlikely if all of the aforementioned procedures are strictly adhered to, in case of emergency, a second formaldehyde spill response kit should be purchased and stored at the MRI facility if formalin is being used.
4.3. Place the MRI-compatible brain container (sealed in multiple layers of plastic bags) with the handle/lid-side facing outward on a folded sheet or cylindrical foam pad to reduce vibrations and keep the container in position during the MRI scanning session.
4.4. Install the anterior elements of the head coil around the MRI-compatible brain container (Figure 3). Note: if possible (i.e., if the MRI facility has a large flex coil and the system has enough receiver channels to accommodate it in addition to the head coil), we have found that it is also beneficial to strap, tape, and/or brace a large 4-channel (knee) flex coil over any potions of the brain container extending beyond the inferior portion of the head coil (i.e., since the lid and handle extend slightly beyond our 12-channel Siemens head coil).
4.5. Acquire a series of localizer scans to: (1) check for severe artifacts arising from bubbles in the brain and/or MRI-compatible container, (2) properly orient the brain, and (3) assign slice positions for subsequent data acquisition.
4.6. Acquire high-resolution, whole-brain, *ex vivo* MRI data (hopefully without magnetic susceptibility artifacts from air bubbles).

### Removing the Brain from the MRI-Compatible Container and/or Changing Formalin

Note: if scanning a brain that has not already been completely fixed, the specimen will either need to be removed from the MRI-compatible container (and placed back in a large formalin fixation bucket) or the formalin in the MRI-compatible container will need to be changed every few days (due to the limited volume of formalin within the container). If this is the case, it is recommended that the following steps be performed.

5.1. Bring the container from the MRI center back to the formalin changing facility and put on proper safety equipment (see above in step 3.4).
5.2. In the fume hood, remove the polyethylene and polypropylene bags surrounding the MRI-compatible brain container with sharp scissors.
5.3. Following the procedures outlined in step 3.2, attach a 5-lb weight to the bottom of the MRI-compatible brain container, place it in a large polyethylene bag, and then gently insert it into the corresponding mold in the large bucket.
5.4. Slowly unscrew the lid of the container and remove the cotton from the top of the container.
5.5. If removing the brain for long-term storage or follow-up histopathology, very gently lift it (with two hands) into a larger, long-term storage bucket that has been filled with fresh formalin and dispose of the used formalin according to steps 3.16–3.18 above.
5.6. If changing the formalin and keeping the brain in the MRI-compatible container, please follow steps 5.7–5.9 below.
5.7. Carefully pour the used formalin (using a funnel) into a chemical Baritainer that has been specifically marked for formalin waste. Note: if two people are involved, this can be done without removing the brain from the MRI-compatible container (i.e., while one person pours the formalin, the other one gently holds the brain from sliding out of the container). However, if working alone, the brain should be temporarily removed from the MRI-compatible container and placed into a separate bucket of formalin.
5.8. Once all of the used formalin has been removed, new formalin can be poured into the MRI-compatible brain container following the procedures outlined in steps 3.4–3.19.
5.9. Then, add the proper amount of formalin-neutralizing compound to the Baritainer, and wait for the specified amount of time before discarding the neutralized formalin, according to steps 3.16–3.19 above.

## Anticipated Results

**Figure 4:**
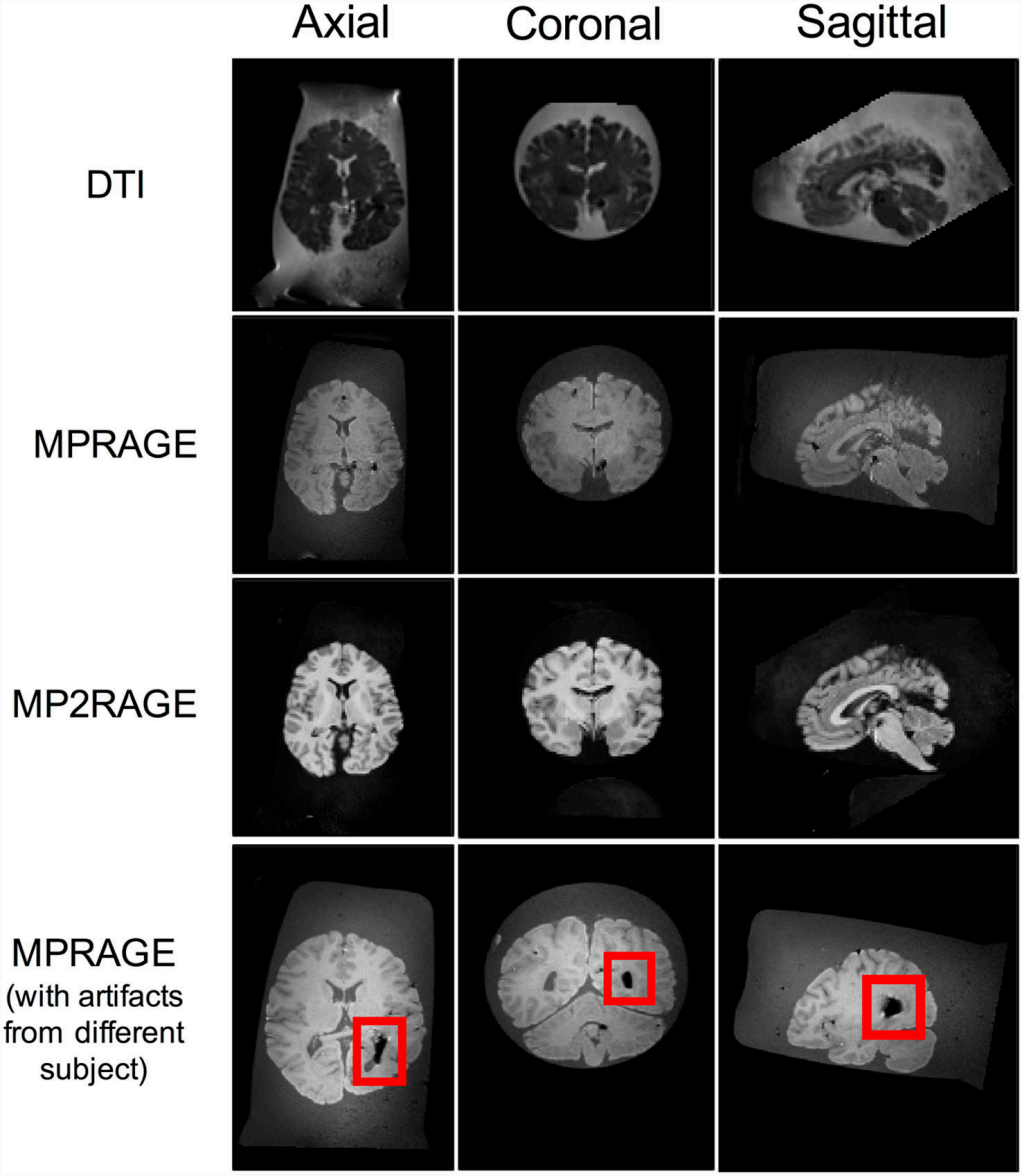
**Axial, coronal, and sagittal slice views of a 71-year-old female brain acquired using the aforementioned MRI sequences following the protocols of this report**. The image artifact (shown in red box) was caused by air bubbles inside the ventricle.

In order to illustrate the image quality that can be obtained following the aforementioned protocol, two whole human brains were scanned at room temperature (~22°C) using a whole-body 3-T Siemens Magnetom Verio scanner (Siemens Healthcare, Erlangen, Germany), equipped with a standard 12-channel head coil and a large 4-channel (knee) flex coil to ensure adequate/uniform signal across the entire field of view (FOV) of the MRI-compatible brain container (Figure 3). For the first brain, the optional procedure in step 3.8 was performed (i.e., to inject the ventricles with formalin), while this option was skipped for the second brain sample (i.e., to show the types of artifacts that can result from air trapped within the ventricles) (Figure 4).

Magnetic resonance imaging data were acquired from each brain after ~168 h of formalin fixation, and were scanned in a formalin-filled container. Representative data were acquired using three different pulse sequences (Figure 4), including: (1) a spin-echo EPI DTI sequence (33) with isotropically distributed diffusion-weighting along 30 directions (*b* = 700 s/mm^2^, plus 5 *b* = 0 s/mm^2^ images), TR = 8800 ms, TE = 73.6 ms, flip angle = 180°, number of slices = 55, FOV = 240 mm × 240 mm, spatial resolution = 1.25 mm × 1.25 mm × 2.5 mm, number of averages = 2, acquisition time = 10.27 min; (2) a 3D T1-weighted magnetization prepared rapid gradient echo (MPRAGE) (28) with TR = 1900 ms, TE = 2.49 ms, TI = 900 ms, flip angle = 9°, FOV = 250 mm × 250 mm × 176 mm, spatial resolution = 0.49 mm ×49 mm × 0.98 mm, number of averages = 1, acquisition time = 4.40 min; and (3) a 3D MP2RAGE, which is a self bias-field corrected sequence for improved segmentation and T1-mapping at high field (34) with TR = 5000 ms, TE = 2.87 ms, TI_1_ = 700 ms, TI_2_ = 2500 ms, flip angle = 5°, FOV = 234 mm × 256 mm × 176 mm, spatial resolution = 1.82 mm × 1.82 mm × 1.00 mm, number of averages = 1, and acquisition time = 5.28 min.

Overall, the results obtained after using this technique were excellent – especially after injecting the ventricles with formalin. A sample of each image type is shown below in Figure 4, where it can be seen that even in the DTI data – which are generally sensitive to even small magnetic susceptibility distortions (35) – the images appear sharp and artifact-free. However, the bottom row shows what can happen if air gets trapped in the lateral ventricles and the optional water/formalin injection is not performed in step 3.8.

## Discussion

Although there are many potential sample preparations for *ex vivo* MRI of human brain tissue, more *ex vivo* studies have been done on fixed brain slices, tissue sections, or hemispheres than on whole human brains. However, there are several obvious advantages to scanning whole brains, especially for exploratory imaging (e.g., to guide subsequent pathological examinations), examining diffuse pathologies, or for things such as whole brain volumetric analyses. Therefore, the purpose of this paper was to introduce an easy and affordable protocol for conducting *ex vivo* MRI experiments on whole human brains.

This protocol addresses many of the limitations reported in previous *ex vivo* imaging studies, including: air cavities/bubbles, motion sensitivity, high cost, and the potential need to regularly change fixative solution. Air bubbles, in particular, are a common and significant problem for *ex vivo* image acquisition if not removed properly. Hence, the initial localizer MRI images should be checked, and if air bubbles are observed inside the ventricles or along the cortical surface, the scan should be stopped until the problem can be resolved (since information in those regions will be compromised in subsequent scans as well).

Motion is another problem that needs to be properly addressed. Previous studies have embedded brains or brain tissue in agarose gel – either in a rectangular container (18), plexiglass cradle (26), or cardboard frame (12) – to ensure stability while scanning. While this approach is likely very effective for controlling bulk motion, it does not isolate table vibrations that are quite prominent during certain types of scanning (e.g., diffusion imaging); plus, embedding in agarose prior to scanning may not be possible (e.g., depending on when in the fixation process scanning needs to occur, what follow-up procedures clinical pathologists need to perform after scanning, etc.). However, although we suggest using cotton bats at the top and bottom of the brain (inside the MRI-compatible container) and a foam pad (outside the container) in an attempt to resist different kinds of motion during the scans – not to mention the fluid barrier inside the container – these measures may not completely eliminate bulk motion or scanner-related vibrations either. Therefore, we still recommend that proper care be taken during subsequent image processing (e.g., applying motion-correction algorithms, etc.).

Although the proposed *ex vivo* scanning preparation has several advantages (discussed above), the 1-gallon (3.8 L) container presents a couple of potential limitations. First, this preparation has been optimized for human MRI systems and the container will not fit in most small-bore 7, 9.4, or 11.7 T preclinical imaging systems. Second, we have shown that the container fits very well within the standard 12-channel Siemens head coil, but coils with different configurations (e.g., Siemens 32- and 64-channel coils) and coils from other manufacturers/vendors (e.g., Philips, GE, etc.) may not accommodate the container. Therefore, before using this setup with other coils, the container should be tested ahead of time to ensure that it will physically fit. If it does, then it should be centered as much as possible between the anterior and posterior elements of the coil to produce the most uniform SNR profile throughout the brain; otherwise (if it will not fit), surface coils could be used as an alternative. However, if surface coils are being considered, it is perhaps worth noting that reducing the distance between the coil and the brain sample (e.g., using a smaller, more conformal, and/or thinner-walled sample container) would improve the SNR of the resulting images, all else being equal.

Finally, it should be emphasized that the polyurethane-based insulating foam, plastic epoxy, and formalin are toxic, and that the brain specimens themselves are a significant biohazard risk. Proper care should therefore be taken when working with these materials. As a rule, we would recommend that anyone planning to replicate our procedure should: (1) read the MSDS and (2) carefully review all of the manufacturers’ instructions before handling these or any other chemicals they plan to work with. Moreover, we would recommend that at least two people are present at all times during these procedures.

In conclusion, *ex vivo* human brain MRI can provide useful information on both the macroscopic/anatomical and microscopic/microstructural level; however, proper sample preparation is an important component for achieving good image quality. Although the quality of *ex vivo* MRI data also depends on other factors (i.e., scanner hardware, image acquisition parameters, image pre- and post-processing, etc.), none of these matter if the images are degraded by magnetic susceptibility artifacts. Therefore, the steps described in this report should help both clinicians and researchers easily and economically acquire high-quality, artifact-free *ex vivo* MR images of whole postmortem human brain samples.

## Author Contributions

AS and CF designed the protocol with contributions from KM. AS, CF, and KM performed the experiments. AS analyzed the results. AS wrote the manuscript with contributions from CF and KM.

## Conflict of Interest Statement

The authors declare that the research was conducted in the absence of any commercial or financial relationships that could be construed as a potential conflict of interest. The reviewer DR and handling Editor declared their shared affiliation, and the handling Editor states that the process nevertheless met the standards of a fair and objective review.

## Footnotes

^1^Note that an adult human brain weighs approximately 3.3 lbs and has a volume of approximately 1130 cm3 for women and 1260 cm3 for men (31).
